# Atypical drainage of a persistent left vena cava superior into the left atrial appendage detected by multidimensional imaging: a case report and review of the literature

**DOI:** 10.1186/s13256-021-03210-9

**Published:** 2021-12-14

**Authors:** Sophie Lengning, René Aschenbach, P. Christian Schulze, Marcus Franz

**Affiliations:** 1 Department of Internal Medicine I, University Hospital Jena, Am Klinikum 1, 07747 Jena, Germany; 2 Institute of Diagnostic and Interventional Radiology, University Hospital Jena, Jena, Germany

**Keywords:** Persistent left vena cava superior, Atypical drainage, Left atrial appendage, Multidimensional imaging

## Abstract

**Background:**

While it is the most common thoracic venous anomaly, a persistent left vena cava superior may present in atypical variations, which are important to consider during clinical management.

**Case presentation:**

Here we report a 35-year-old Caucasian female patient with drainage into the left atrial appendage who presented with shortness of breath accompanied by mild hypoxemia. Venous contrast filling in the context of pulmonary scintigraphy suspected an additional superior caval vein connected to the left atrial appendage. Diagnosis was confirmed by transesophageal echocardiography. Cardiac catheterization revealed a minor right-to-left shunt. The symptoms could be allocated to a bronchial asthma and treated according to guidelines. Cerebral lesions detected in the patient were due to a coincident multiple sclerosis rather than cerebral embolisms. Thus, the venous anomaly was classified as an incidental finding currently requiring no treatment.

**Conclusions:**

To the best of our knowledge, this is the first report of a persistent left vena cava superior draining into the left atrial appendage.

**Supplementary Information:**

The online version contains supplementary material available at https://doi.org/10.1186/s13256-021-03210-9.

## Background

Being the most common venous anomaly of the thorax, a persistent left vena cava superior (PLVCS) draining into the right atrium comprises up to 90% of cases and is mostly asymptomatic. Drainage into the left atrium, causing a right-to-left shunt is rare, but may cause severe hypoxemia and presents a risk for embolic brain lesions. Its usual drainage location has been allocated between the pulmonary veins and the base of the left atrial appendage [1], with atypical variants as presented here:

A multimodal imaging and quantification approach identified a PLVCS draining into the left atrial appendage. To the best of our knowledge, this is the first report displaying this anomaly. There is one further case report from 1982 describing a patient with complex congenital heart disease including a PLVCS draining into the right atrium via coronary sinus. Additionally, this PLVCS was connected to a rudimentary left atrial appendage, which was not connected to the left atrium [2].

With increasing options for surgical and percutaneous correction of the venous anomaly, it is crucial to differentiate this finding from other underlying causes, as presented in this case: Hypoxemia could be assigned to the pulmonary focus of a bronchial asthma, and cerebral lesion to a multiple sclerosis rather than desaturation and multiembolic events due to the right-to-left-shunting.

## Case presentation

### Clinical presentation

A 35-year old Caucasian female with a history of multiple sclerosis and atopic diathesis (including bronchial asthma) presented for chronic respiratory discomfort to obtain a second opinion on her treatment. Furthermore, she had noted recurrent coughing that deteriorated in autumn. Prednisolone prescribed due to her allergic asthma had only insufficiently relieved symptoms. Previously, she had completed a specific immunotherapy addressing her allergy towards certain phytocomponents, and quit smoking after accumulation of 2 pack years in total. With seasonal fluctuation of symptoms, spirometry revealed stable static and dynamic respiratory flow values with slight obstruction. Asthma control testing (ACT), a patient-based questionnaire [3], revealed eight points at initial presentation (target range ≥ 19 points), indicating poorly controlled asthma. There were no signs of acute infection and an influenza immunization had recently been implemented.

Next to oral contraception and seasonal antihistamines, her medication included inhalative steroids in combination with a long-acting betamimetic agent and a short-acting betamimetic nebulizer on demand. Therapy with montelukast had to be discontinued due to gastrointestinal adverse effects. Her relapsing–remitting multiple sclerosis was kept in a steady state by dimethyl fumarate (Tecfidera) and glatiramer acetate. She experienced two episodes in the past 9 years and was left with residual mild hypoesthesia, gait disturbances, and chronic fatigue.

Physical examination detected no pathologies in the patient, a medium-build [body mass index (BMI) 21 kg/m^2^] woman in good condition. Auscultation revealed vesicular respiratory sounds without rales. No cardiac murmur was audible. Her fingers did not show clubbing. Cardiac natriuretic peptides presented within normal limits. Besides hypereosinophilia (0.27 GPL/L), laboratory testing revealed a hypoxemia with an adequate response to oxygen (initial: 67 mmHg, with 3 L O^2^: 72 mmHg), that persisted in rest and exertion.

Due to this blood gas analysis presenting relevant hypoxemia, further investigations were undertaken. In the meantime, daily self-assessment of peak flow values for continuous monitoring of dynamic respiratory function was recommended.

### Investigations

Scintigraphy was performed to rule out a ventilation perfusion deficiency. For the subjacent computed tomographic (CT) scan, contrast media was injected into the left brachial veins. Secondarily to the exclusion of pulmonary embolism, venous contrast filling suspected an additional left superior cava vein connected to the left atrium.

**Fig. 1:**
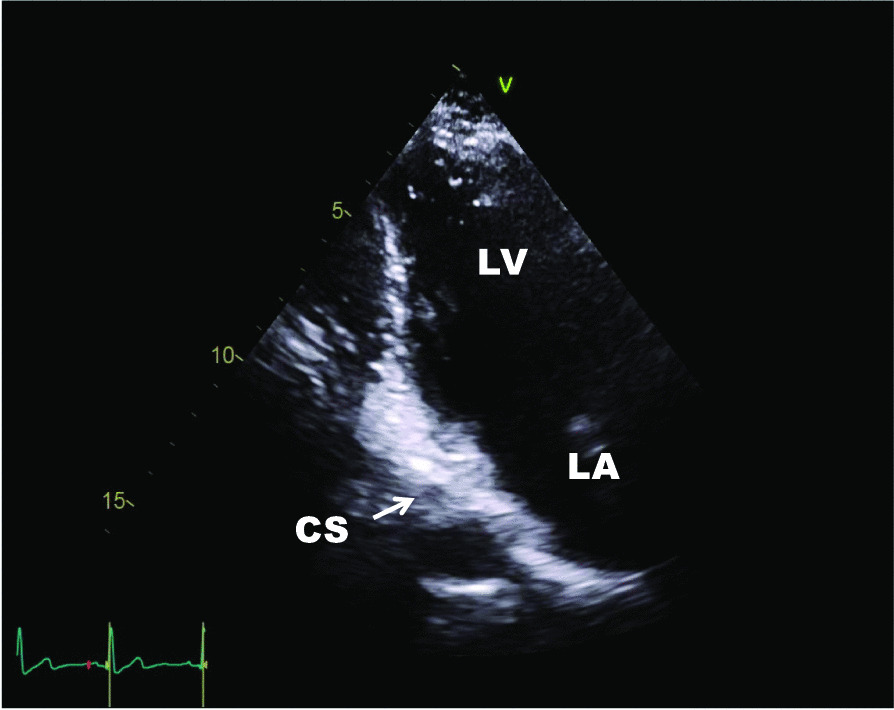
Transthoracic two-chamber view displaying the left side cavities (LA left atrium, LV left ventricle) without evidence of a dilated coronary sinus (CS, white arrow)

**Fig. 2:**
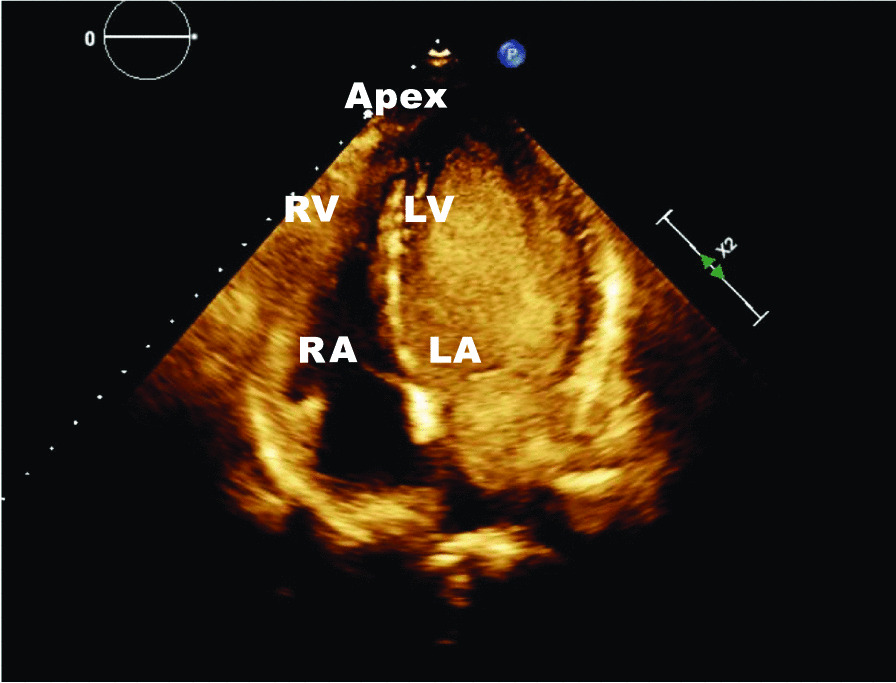
Apical four-chamber view shows solely an opacification of the left heart (*LV* left ventricle, *LA* left atrium) without contrast media affecting the right sided cavities (*RV* right ventricle, *RA* right atrium)

**Fig. 3:**
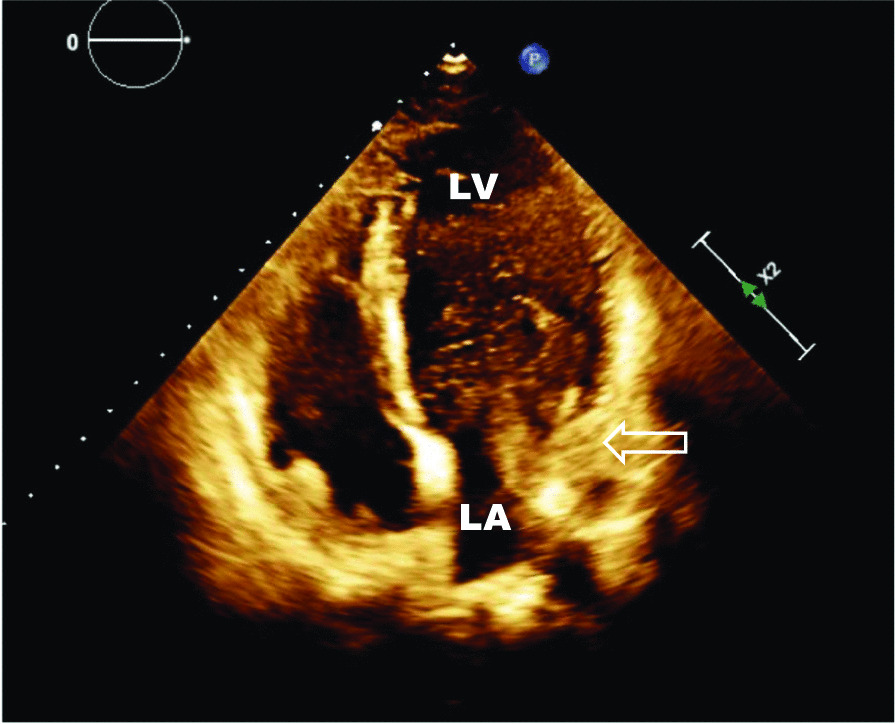
Contrast media injected into the left brachial vein enters the left atrium (LA) on its lateral base—as indicated by arrow (←)—where the left atrial appendage is located just anteriorly

**Fig. 4:**
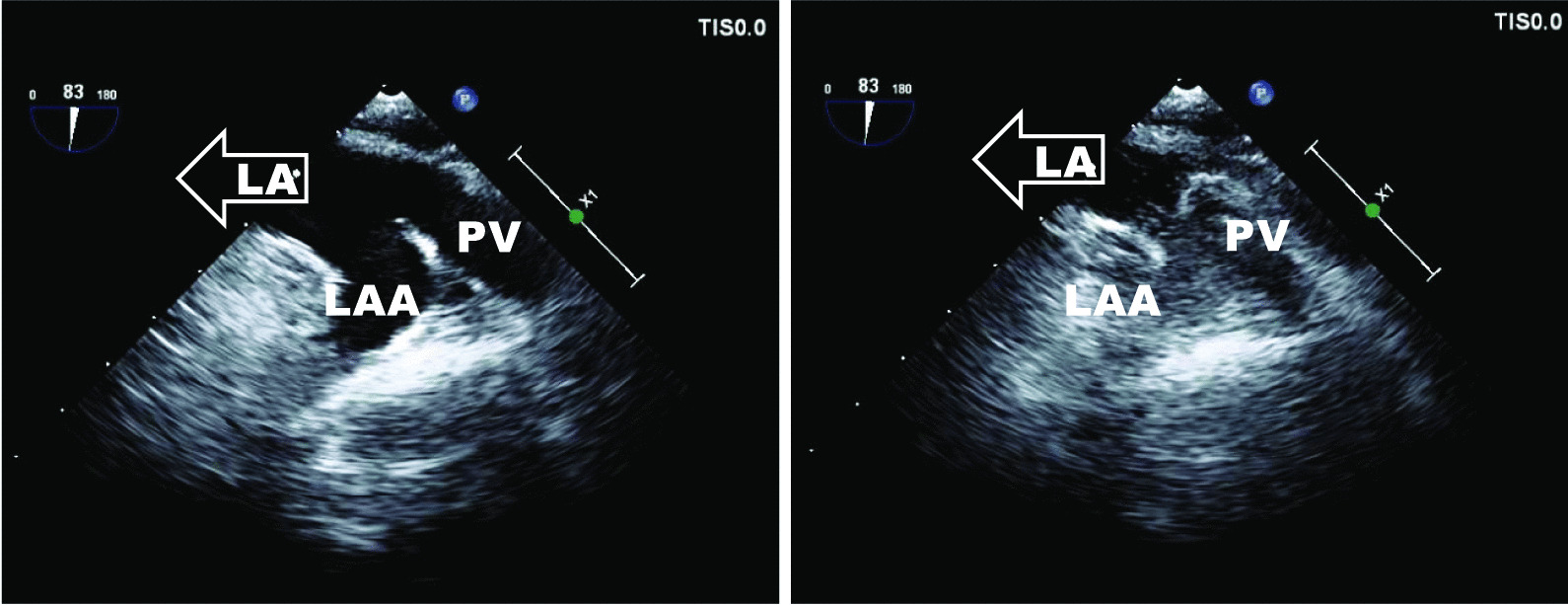
Transesophageal view at the junction of left atrial appendage (LAA) and left pulmonary veins (PV) before inflow into the left atrium (*LA*; left image). The contrast media inflow origins in the left atrial appendage where the opacification commences (right image)

**Fig. 5:**
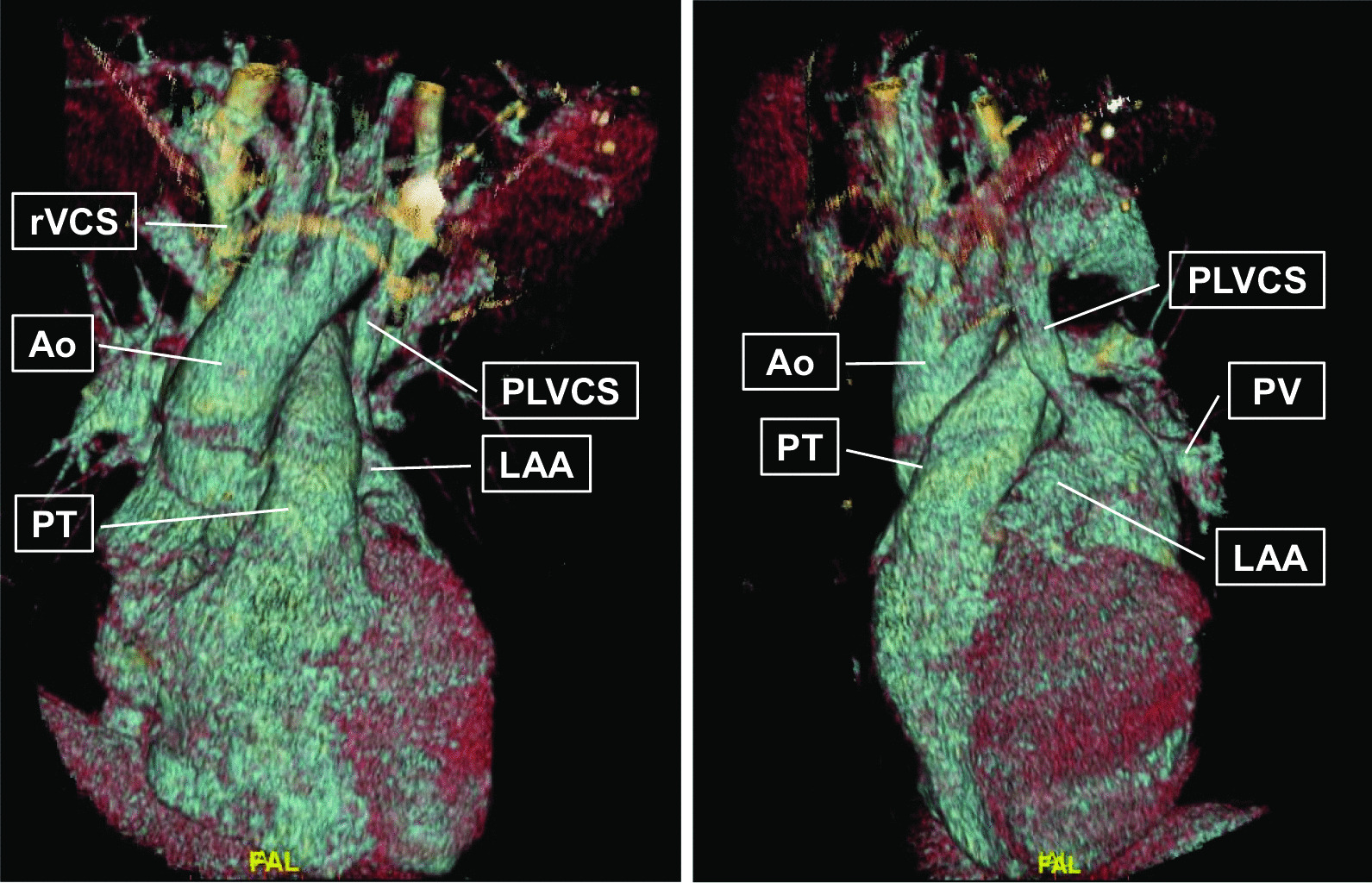
Reconstructed CT imaging reveals the atypical drainage of the persistent left vena cava superior (PLVCS) into the left atrial appendage (LAA). The lateral view (left image) exposes a more precise view this rare anatomical variant, while the anterior view (left image) displays the bilateral superior caval vein (*rVCS* right vena cava superior). *Ao* Aorta, *PT* Pulmonary truncus

The diagnosis was confirmed by echocardiography: following the attestation of a normal biventricular function with regular sized atria and no evidence of dilatation of the coronary sinus (Fig. 1), contrast media was applied via the left brachial veins. Recordings of the apical four chamber view demonstrated an instant and complete opacification solely of the left-sided cardiac cavities (Fig. 2). The right atrium and ventricle were unaffected and septal structures were intact. Contrast media inflow originated from the lateral base of the left atrium (Fig. 3). On transesophageal echocardiography (TEE), contrast enhancement commenced at the junction of the left atrial appendage and the left pulmonary veins as depicted in midesophageal pulmonary vein view (Fig. 4). As the CT scan reconstruction visualizes (Fig. 5 and Additional file 1: Video S1), the drainage location site could be specified as being the left atrial appendage. Herein, the persistent left vena cava superior complied with type IIIb of Schummer’s classification of PLVCS, with an atypical draining into the left atrial appendage [4].

Following the imaging modalities, cardiac catheterization was performed to assess the effect of the PLVCS on the patient’s hemodynamics. Pulmonary circulation showed normal proportions of pressure and resistance. The composition of intracardiac blood gas analyses revealed a Qp/Qs of 1.2, reflecting a hemodynamically nonrelevant right-to-left shunt, with an oxygen saturation of 69% in the PLVCS not altering the saturation of the left atrium (95%). To assess its functional impact, we complemented a spiroergometry. The patient completed the testing with a peak oxygen uptake of 23 mL/min/kg, rated as an unrestricted exercise capacity.

Furthermore, the cerebral condition was—on account of her multiple sclerosis—frequently appraised by magnetic resonance imaging. T2-weighted sequences revealed multiple lesions of varying sizes in various cerebral compartments, mainly in white matter (frontal, mesencephalic, occipital) and cerebellar.

### Differential diagnosis

The main diagnostic concerns in the present case of an atypical draining PLVCS into the left atrial appendage were to distinguish the hypoxemia and neurologic disorders from the known bronchial asthma and multiple sclerosis.

The initial complaints about dyspnoea on exertion and coughing were accompanied by mild hypoxemia. After imaging revealed a PLVCS draining atypically into the left atrial appendage, further testing was implemented. The resulting right-to-left-shunt that led to an opacification of the left atrium during echocardiography could have been the etiology. Invasive testing revealed an oxymetrically nonrelevant shunt, which later allowed unrestricted exercise testing. In the meantime, the patient received bronchial asthma treatment according to guidelines. She especially stated a relief of her symptoms using inhalative tiotropium bromide in addition to betamimetics and physical conditioning. The frequency of her coughing attacks declined using steam inhalation with brine. Thereupon her asthma control testing increased from 8 to 19 points (the latter being within physiological limits), indicative of a correlation between symptoms and asthma rather than the incidental finding of PLVCS.

Regarding her cerebral lesion, a differentiation between injuries from her known multiple sclerosis and paradoxical embolic insults from her right-to-left-shunt was essential. The patient had developed symptoms suspicious of multiple sclerosis for 9 years at the time of presentation: chronic fatigue, mild hypoesthesia, gait disturbances, and voiding dysfunction, but no focal deficiency. These symptoms resided from two episodes of her relapsing–remitting disease progress analogous to temporal dissemination. Magnetic resonance imaging (MRI) revealed a demyelination mainly of white matter and cerebellar structures, as represented in the reviewed McDonald criteria. Responsive to immunomodulating treatment with dimethyl fumarate and glatiramer acetate, clinical and imaging follow-ups over the past 5 years have not shown disease progression, with a constant disability score (EDSS) of 3.5. Though T2-hyperintense, the lesions showed no relevant signs of infarction (cortical location, diffusion deficiency, contrast enhancement). Typical symptoms, time course, imaging, and response to specific treatment assigned her neurologic disorders to multiple sclerosis rather than embolic strokes resulting from right-to-left-shunting caused by PLVCS.

### Treatment and outcome

Proposed treatment options of a persistent left vena cava superior with drainage into the left atrium include percutaneous closure with an Amplatzer device [5] and surgical intervention, with the latter usually being subject to complex operations of coexisting cardiovascular defects [6]. These interventions are reserved for symptomatic patients.

The presented case of an atypical drainage into the left atrial appendage resulted in a minor right-to-left-shunt. Its subordination correlated well with adequate oxygen utilization, quantified by spiroergometry. The left atrium showed no evidence of volume overload. Substantiated with a small shunt volume and no evidence of embolization, no specific treatment was required for the venous anomaly. This restraining approach is subject to constant reevaluation in case of an aggravation.

The respiratory symptoms were allocated to a known and lately aggravated bronchial asthma that was successfully treated with inhalatives according to current guidelines.

Since no specific treatment was required, further therapy of the coexisting medical conditions will reside with the respective practitioners. Nonetheless the patient will remain in cardiologic control for periodic surveillance at regular intervals.

## Discussion

Technical advancement, particularly regarding imaging modalities, increases the detection of unexpected anatomical variations. This includes the persistent vena cava superior that usually finds its way to the right atrium. In the absence of further cardiac defects, PLVCS is likely to be asymptomatic. Prevalence in the general population is around 0.3% [7]. However, the prevalence is higher in the pediatric population, emphasizing its association with other, more complex cardiac anomalies (e.g., heterotaxy) [8]. Embryologically, a failure in regression of the anterior cardinal vein results in persistence of the left superior vena cava [7]. During normal fetal development, it is supposed to obliterate and form the coronary sinus, substantiating the relatively usual finding of bilateral vena cava with the PLVCS draining into the right atrium via the dilated coronary sinus. The latter is predisposed to be the index finding that leads to the detection of PLVCS [8]. In around 10% of cases, atypical draining into the left atrium occurs, which is associated with serious hazards. Causing an intracardiac shunt, this presents a risk for septic or thrombotic brain lesions secondary to left-to-right shunting with paradoxical embolization [7, 9]. Moreover, a large shunt volume predisposes to hypoxemia [10]. A previously published case series of patients with atrial fibrillation where a PLVCS may act as a driver or trigger describes two patients (0.05%) with a drainage to the left atrium [11]. PLVCS are described as encountering the left atrium between the pulmonary veins and the base of the left atrial appendage [1].

The current case displays a very rare variant of this anomaly. The patient presented with chronic shortness of breath and coughing, accompanied by mild hypoxemia. Multimodal imaging revealed not only a PLVCS but a surprising drainage location: into the left atrial appendage. Due to the possible life-threatening complications stated above, further investigations were prompted. Oxymetrical testing revealed a minor shunt volume and dyspnoea could be assigned to an underlying bronchial asthma. Since cerebral imaging displayed multiple lesions, embolism secondary to right-to-left-shunting was suspected as described in other patients [7, 9]. Subsequent investigations assigned the cerebral lesions to a multiple sclerosis, leaving this exceptional isolated PLVCS an incidental finding. With percutaneous and operative closure techniques obtainable, no specific treatment was required [5]. It has been reported the left atrial appendage itself may serve as patch material in surgical redirection of PLVCS into the right atrium [12].

To the best of our knowledge, this is the first case report describing a PLVCS draining directly into a normally developed left atrial appendage.

## Conclusions

Knowledge of a PLVCS—especially when draining into the left atrial appendage—may have further implications in the future, for example, in treatment or risk scoring of a contingent atrial fibrillation or if an LAA occlusion becomes necessary. Furthermore, pacemaker-led or central venous catheter implantation may not only be complicated by a PLVCS but also lead to serious consequences [13]. Since the drainage into the left atrial appendage is a very rare variant of PLVCS, it may affect the embolic risk, especially in coexisting atrial fibrillation.

## Supplementary Information

**Additional file 1: Video S1. **Reconstructed CT imaging reveals the atypical drainage of the persistent left vena cava superior (PLVCS) into the left atrial appendage (LAA). The lateral view (left image) exposes a more precise view this rare anatomical variant while the anterior view (left image) displays the bilateral superior caval vein (*rVCS* right vena cava superior). *Ao* Aorta, *PT* Pulmonary truncus.

## Data Availability

Not applicable.
